# Supplementary figures and images for: Diagnostic system for the detection of severe fever with thrombocytopenia syndrome virus RNA from suspected infected animals
Source: PLoS One. 2021 Jan 28;16(1):e0238671. doi: 10.1371/journal.pone.0238671 (PMC7842937; doi:10.1371/journal.pone.0238671)

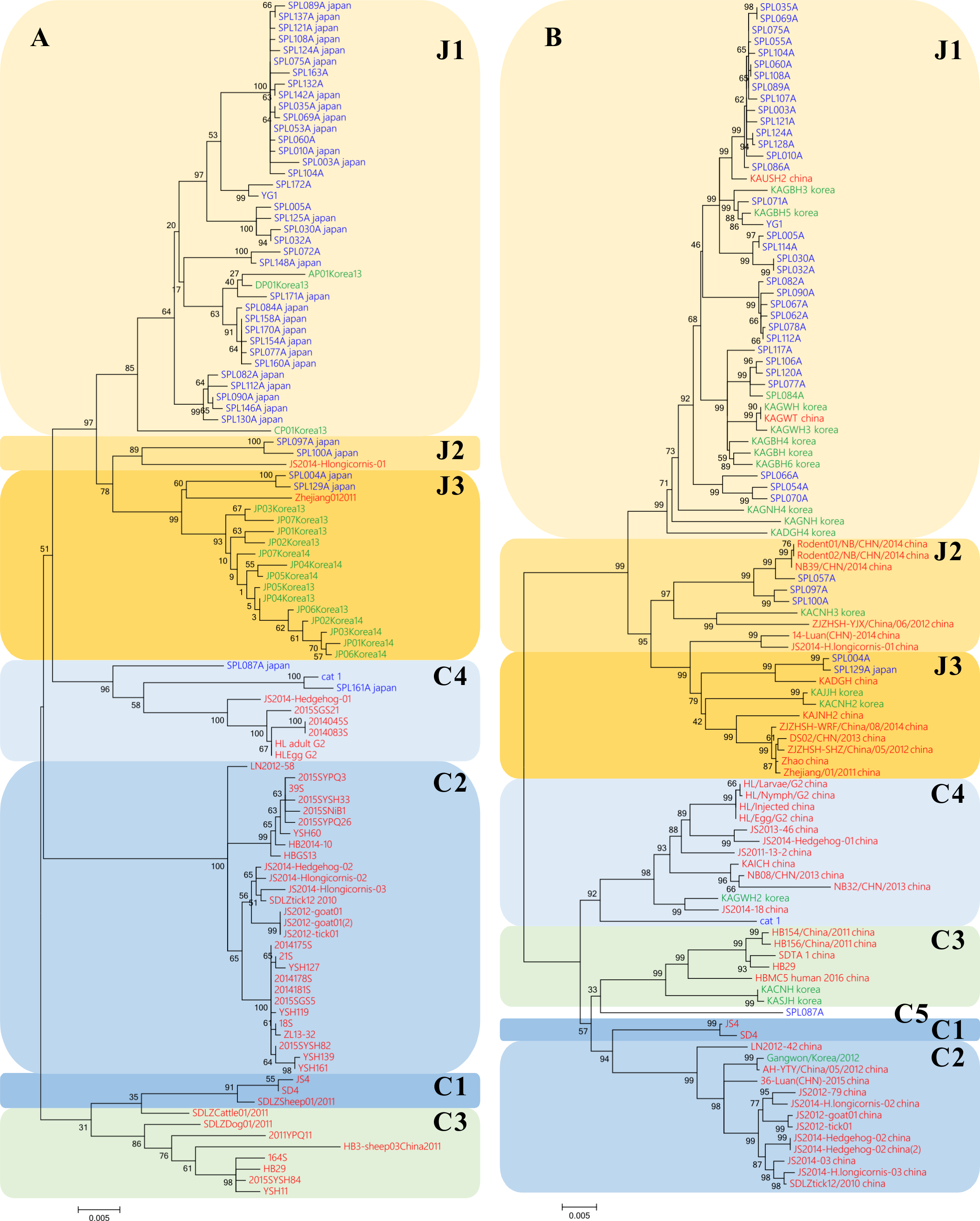

Supplement: S1 Fig — Strains that were identified in China, Japan and Korea are indicated by red, blue and red, respectively. (TIF) [file pone.0238671.s001.tif]
