# Supplementary material for: Diagnostic system for the detection of severe fever with thrombocytopenia syndrome virus RNA from suspected infected animals
Source: PLoS One. 2021 Jan 28;16(1):e0238671. doi: 10.1371/journal.pone.0238671 (PMC7842937; doi:10.1371/journal.pone.0238671)
Supplement: Attachment — Submitted filename: Response to Reviewers.docx [file pone.0238671.s003.docx]

Minor points

1. Line 15: “those of SFTS patients, whereas SFTS-contracted cats….”

Response : We corrected the sentence according to the comment (line 15-16).

2. Line 22: “specifically detected six clades of SFTSV” in the Abstract was not shown in the Result section. Please consider the revision.

Response : “six clades” were changed to “four clades” since two clades were not included in the clinical samples in the present study. However, the primers designed in the completely conserved region among eight clades, thus the PCR might detect all the eight clades. In this regard, we added the contents in the Result and Discussion section (line 173-174, 192-194).

3. Line 35: “indicating the cat-to-human transmission of SFTSV”?

Response : The sentence was corrected according to the comment (line 36).

4. Line 46: a variety of animals, while viral RNA and antibodies…”

Response : The sentence was corrected according to the comment (line 46).

5. Line 64: “vomiting”

Response : The sentence was corrected according to the comment (line 67).

6. The rationale of new RT-PCR appeared in Discussion (lines 182 – 187). A similar brief rationale should be given in Introduction and Abstract sections.

Response : The brief rationale were added in the Abstract and Introduction sections (line 18-19, 53-55).

7. Title: “Diagnostic system for the detection of severe fever with thrombocytopenia syndrome virus RNA from suspected infected animals” or “Diagnostic system for the RNA detection of severe fever with thrombocytopenia syndrome virus from suspected infected animals”

Response : Title was changed to “Diagnostic system for the detection of severe fever with thrombocytopenia syndrome virus RNA from suspected infected animals”.
